# Social Support, Posttraumatic Stress Disorder and Growth Among Adolescents During Public Health Emergencies: Mediating Roles of Empathy and Coping Styles

**DOI:** 10.3390/bs16030377

**Published:** 2026-03-06

**Authors:** Baohua Zhen, Benxian Yao, Rui Zhen

**Affiliations:** 1 School of Psychology, Nanjing Normal University, Nanjing 210024, China; zbh3399@126.com; 2 College of Educational Science, Anhui Normal University, Wuhu 241000, China; 3 Jing Hengyi School of Education, Hangzhou Normal University, Hangzhou 311121, China; zhenrui1206@126.com

**Keywords:** social support, empathy, coping styles, PTSD, PTG

## Abstract

Public health emergencies can trigger posttraumatic stress disorder (PTSD) and posttraumatic growth (PTG) in adolescents. However, few studies have explored the distinct and common processes of these outcomes in adolescents from the perspective of social support during public health emergencies, and whether the mechanisms underlying these phenomena are unique or shared remains unclear. This study examined how social support relates to PTSD and PTG, with empathy, positive coping, and negative coping as mediators. A cross-sectional study using self-report questionnaires collected data from 921 Chinese junior middle school students. The results showed that social support was directly negatively associated with PTSD and positively associated with PTG. Social support was negatively associated with PTSD via positive coping styles (PCSs), negative coping styles (NCSs), and through a two-step path from empathy to PCSs. Social support was negatively associated with PTG via NCSs, and positively associated with PTG via empathy, PCSs, and through a two-step path from empathy to PCSs. Findings suggest partly distinct pathways linking social support to PTSD and PTG: empathy was related to PTG but not PTSD, PCSs functioned as a shared pathway, and NCSs showed a double-edged pattern. Parents and teachers should foster adolescents’ empathy and PCSs to promote healthy psychological development after public health emergencies.

## 1. Introduction

Public health emergencies, such as COVID-19—one of the most significant public health emergencies in recent years—can lead to substantial physical harm and traumatic impacts on mental health (Chamaa et al., 2021). Research indicates that many people who encountered the COVID-19 pandemic later manifested psychological problems (Wolf & Schmitz, 2024). One of the most frequently reported psychological reactions is posttraumatic stress disorder (PTSD) (Yunitri et al., 2022). Importantly, adolescents tend to exhibit a higher risk of psychological problems compared with adults because their cognitive and emotional regulatory capacities have not yet matured (Clark et al., 2020). A meta-analysis by Ma et al. (2021) indicated a 26–48% prevalence of assorted mental health issues among adolescents during COVID-19, a period in which Selçuk et al. (2021) specifically identified PTSD symptoms in 28.5% of adolescents. However, exposure to potentially traumatic events does not only result in negative psychological outcomes or symptoms. In some cases, it can act as a catalyst for positive psychological transformation, particularly among adolescents (Tedeschi & Calhoun, 2004). When individuals cope with the negative effects of a traumatic event, growth in life philosophy, self-identity, and relational intimacy may emerge, a process known as posttraumatic growth (PTG). Previous studies have reported that 45.6% of adolescents showed varying degrees of PTG during a public health emergency (J. Zhou et al., 2021).

The high prevalence rates of PTSD and PTG raise a critical question of whether these two posttraumatic outcomes arise from the same underlying mechanisms in the same person. Previous research indicates that PTSD may facilitate the emergence of PTG (Dekel et al., 2012), and PTSD and PTG may share common causal mechanisms (Dekel et al., 2011; X. Zhou & Zhen, 2024). Such evidence is consistent with the possibility that PTSD and PTG may co-occur following trauma (Wu et al., 2018). Nevertheless, although the prerequisites for the occurrence of PTSD and PTG appear similar, their underlying processes may differ (X. Zhou et al., 2022). Moreover, while existing research has largely focused on cognitive factors and personal resources (Y. Zhang et al., 2019), external resources, such as social support, also play a crucial role in shaping individuals’ traumatic psychological reactions (Wu et al., 2018). Although social support, as a paramount external resource, has received attention in prior research (Ning et al., 2023), the mechanisms linking social support to PTG and PTSD among adolescents during public health emergencies remain poorly understood. Therefore, this study aims to clarify both the distinct and common processes through which external resources are associated with PTG and PTSD in adolescents, and to explore whether the mechanisms underlying PTG and PTSD are overlapping or differentiated during public health emergencies.

According to Conservation of Resources (COR) theory (Hobfoll, 2011), experiencing potentially traumatic events may deplete individuals’ resources, thereby threatening their physical and psychological safety, and lead to various physical and mental reactions. In such contexts, the timely replenishment of internal and external resources can help individuals cope with trauma, alleviate their negative psychological reactions, and foster positive changes. Among these resources, social support, as a key component of external resources, provides material and psychological support to individuals, supplementing resources consumed in coping with stress (Yu et al., 2022), helping mitigate negative emotional responses caused by trauma and catalyzes positive adaptation (X. Zhou et al., 2017a). Empirical studies have consistently linked higher perceived social support to lower severity of PTSD symptoms (Dai et al., 2016) and higher levels of PTG (Gök & Çiftçi, 2023). These findings are further corroborated by meta-analysis evidence (Ning et al., 2023; Zalta et al., 2021). Therefore, high levels of social support can directly reduce PTSD and foster the development of PTG.

Importantly, social support may not only directly relate to PTSD and PTG but may also be indirectly associated with them by influencing adolescents’ cognitive and emotional resources. The three-phase process model proposed by X. Zhou and Zhen (2024) suggests that the development of PTSD and PTG unfolds across three phases—emergency, coping, and reaction—within which social support, cognitive, and emotional factors all play important roles. Among these factors, social support may influence individuals’ cognitive and emotional resources (Hobfoll, 2011), which are subsequently associated with PTG and PTSD (Wu et al., 2018). Therefore, empathy, a comprehensive construct integrating both cognitive and affective components (M. H. Davis, 1983), could represent a crucial mechanism linking social support to both PTG and PTSD. Specifically, after traumatic events, social support of sufficient quality and quantity acts as a scaffold, constructing a protective and supportive environment for individuals, enhancing their trust and security toward the external world, helping them maintain sensitivity to others’ emotions and cognition, and promoting the emergence of empathy (Jambon et al., 2019). High empathy enables individuals to recognize traumatic events from others’ perspectives, prompting deliberate rumination on trauma, fostering positive changes, and culminating in PTG (X. Zhou et al., 2019). Beyond its role in perspective-taking, empathy may also help individuals express their emotions, reduce the influence of negative emotions, and alleviate their PTSD. Evidence supports a positive relationship where social support correlates with higher levels of empathy (Qin et al., 2022), while high empathy is linked to lower PTSD (Canevello et al., 2021) and higher PTG (El-Gabalawy et al., 2021). Thus, empathy may function as a mediator through which social support is indirectly associated with both PTG and PTSD.

Coping styles, as important cognitive resources, may be another mediating factor in the relationship between social support to both PTG and PTSD. The process theory of coping (Lazarus, 1993) posits that when individuals appraise demand resources as exceeding their personal resources, they will make cognitive and behavioral efforts to cope with stress, which are influenced by both external environment and internal cognitive factors. Therefore, it is reasonable to hypothesize that social support might compensate for the cognitive resources of individuals with traumatic experience, thereby influencing their coping styles, which in turn affect their posttraumatic psychological reactions. Empirical studies have provided support for this view. For instance, prior research indicates that individuals with high social support are more likely to appraise trauma positively (Su & Chen, 2015), and adopt positive coping styles (PCSs) to solve problems at the source (Rajandram et al., 2011; Shen et al., 2018), which may alleviate the influence of negative emotions, reduce PTSD symptoms (Vernon et al., 2009), and promote PTG (Peters et al., 2021; Schuettler & Boals, 2011). In contrast, individuals lacking social support may resort to negative coping styles (NCSs), such as avoidance and denial (L. Zhang et al., 2021), potentially leading to disengagement in their behavior and mental state (Gil, 2005). This can exacerbate negative emotional experiences and inhibit the recovery process, resulting in long-term persistence and aggravation of PTSD (Peters et al., 2021), while also hindering the emergence of PTG (Brooks et al., 2019). Therefore, social support may indirectly associate with adolescents’ PTSD and PTG through different types of coping styles (Hao et al., 2023).

Empathy and coping styles may mediate the associations between social support, PTSD and PTG, but the relationships between empathy and coping styles themselves are also important. Some studies suggest that empathy is a psychological resource that shapes coping styles, acting as a facilitator of PCSs and an inhibitor of NCSs (Sun et al., 2019). Specifically, highly empathetic individuals demonstrate a greater capacity for cognitive and emotional perspective-taking, which enables them to understand problems more comprehensively and adopt PCSs to solve them. Conversely, individuals with low empathy tend to be more rigid in their thinking and emotions, viewing situations primarily from their own perspective. Thus, such individuals are more prone to utilize self-centered NCSs when coping with problems (Moreno-Manso et al., 2018). Empirical studies have also found that individuals with high empathy tend to use more PCSs and fewer NCSs (Ardenghi et al., 2024; M. Wang et al., 2020). Therefore, empathy may be positively associated with PCSs and negatively associated with NCSs. Given the role of social support in enhancing empathy, high social support may improve adolescents’ empathy, which in turn influencing the use of coping styles and ultimately associated with lower PTSD and higher PTG.

After reviewing relevant theories and empirical findings, we find that, although the three-phase process model (X. Zhou & Zhen, 2024) and COR theory (Hobfoll, 2011) provide useful explanations for why social support is associated with PTG and PTSD, studies simultaneously examining the potential mechanisms linking social support to both posttraumatic reactions via empathy, PCSs, and NCSs remain limited. This gap constrains a mechanistic understanding of how an external resource may be translated into adolescents’ cognitive resources and coping resources, which may relate to PTG and PTSD in different ways. Furthermore, although the three-phase process model suggests that PTSD and PTG may share common prerequisites but differ in subsequent reaction processes (X. Zhou & Zhen, 2024), this viewpoint lacks validation from diverse empirical studies that include social support, empathy, and coping styles. These gaps collectively motivated the design of this study, which aims to provide empirical evidence to these issues by examining differentiated indirect pathways involving empathy and coping styles.

**Figure 1:**
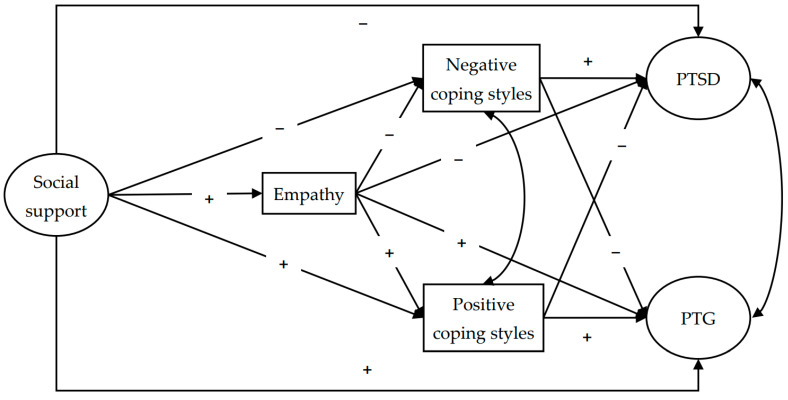
Hypothesized model of mediating effects. Note: A minus (plus) symbol designates a path coefficient that is expected to be negative positive); PTSD = posttraumatic stress disorder; PTG = posttraumatic growth.

Based on relevant theories and empirical findings from previous studies, we constructed the following hypotheses: (H1) social support is positively correlated with PTG and negatively correlated with PTSD; (H2) social support is positively correlated with PTG and negatively correlated with PTSD via empathy, PCSs, and NCSs, respectively; and (H3) social support is positively correlated with PTG and negatively correlated with PTSD through chain mediation from empathy to PCSs, and from empathy to NCSs. Figure 1 illustrates the complete structural model with all hypothesized pathways.

## 2. Materials and Methods

### 2.1. Procedures and Participants

In April 2022, during the ongoing COVID-19 pandemic, adolescent participants were recruited from schools in Hubei Province. Data collection occurred when containment measures were still in place, with school teachers facilitating questionnaire administration while adhering to safety protocols. The final cohort comprised 938 students in grades 7 and 8. No students were recruited from grade 9 because of their demanding graduation schedule. For quality assurance purposes, 17 responses were excluded as invalid because more than 30% of the information was missing or the questions were answered randomly. Ultimately, 921 valid questionnaires were obtained. Males constituted 51.7% of the sample (*n* = 476) and females constituted 45.3% (*n* = 417), while 28 (3.0%) chose not to disclose their gender. Participants’ ages averaged 13.53 years (SD = 0.63), bounded by 12 and 16 years. In terms of grade level, there were 583 seventh-grade students (63.3%), 333 eighth-grade students (36.2%), and five students did not provide their grade information (0.5%). Additionally, most participants were non-only children (781 individuals [84.8%]), while a smaller proportion were only children (115 individuals [12.5%]), and 25 adolescents did not provide this information.

Participants from the chosen classes provided their willing assent to take part and filled out the self-report measures during school hours. Before starting the formal investigation, a professional psychology teacher thoroughly explained the purpose of the research and reiterated that students were free to cease participation at their discretion without repercussions. Students and guardians provided written assent. Trained postgraduate students in psychology supervised the data collection process.

### 2.2. Measures

#### 2.2.1. Social Support

To assess social support, we employed a Chinese adaptation of the Perceived Social Support Scale (PSSS). This scale was adapted into Chinese by Jiang (2001) from the original scale created by Zimet et al. (1988). Three subscales make up the PSSS: family, friend, and other support. The scale has 12 items in total, which are distributed evenly across the three dimensions (four items each). To better align with adolescents’ experiences, as suggested by previous research (Xin et al., 2019), the term “leaders and colleagues” within the dimension of support from others was replaced with “teachers or relatives”. A 7-point rating system was used for responses, where 1 means “strongly disagree” and 5 means “strongly agree”. Adolescents who report higher levels of social support obtain higher total scores on this scale. This part of the research had a Cronbach’s alpha of 0.92.

#### 2.2.2. Empathy

We used the Chinese adaptation (Rong et al., 2010) of the Interpersonal Reactivity Index, a measure originally developed by M. H. Davis (1980). It is composed of four 7-item subscales: perspective-taking, fantasy, empathic concern, and personal distress. The prior literature positions empathic concern within the domain of emotional empathy, focusing on individuals’ attention and affective responses to others’ emotions. Perspective-taking reflects an ability or proclivity to adopt others’ perspectives in real life, and constitutes the cognitive dimension of empathy. In this study, we focused on perspective-taking and empathy concern because they are widely used as core indicators of cognitive and emotional empathy, and they are posited to be strong proxies for assessing general empathy (Siu & Shek, 2005). Fantasy and personal distress were not included because fantasy and personal distress are similar to assessments of individuals’ imagination and emotional self-control, and they may not necessarily serve as intrinsic dimensions for empathy (Baron-Cohen & Wheelwright, 2004). Given our aim to model empathy as a resource-related mechanism linking social support to coping styles and posttraumatic outcomes, we prioritized the two subscales most directly capturing cognitive and emotional empathy. This yielded a 14-item measure, which incorporated five reverse-scored items. A 5-point rating system was used for responses, where 1 indicated “completely inconsistent” and 5 indicated “completely consistent”. Higher scores corresponded to greater empathy. The scale’s reliability was acceptable in the present study, with a Cronbach’s alpha of 0.71.

#### 2.2.3. Coping Styles

The assessment of coping styles was conducted using the Simplified Coping Style Questionnaire, a 20-item instrument created by Xie (1998). The initial 12 items measured adolescents’ PCSs, and the final 8 related to NCSs. Every item was rated on a 4-point scale, with 1 signifying “not adopted” and 4 indicating “frequently adopted”. Elevated scores on a given dimension were interpreted as a greater propensity to employ the respective coping styles. Both subscales demonstrated acceptable reliability (Cronbach’s α = 0.82 for PCSs, 0.71 for NCSs).

#### 2.2.4. PTSD

To evaluate adolescents’ PTSD, we employed the PTSD Checklist for DSM-5 (PCL-5), originally created by Weathers et al. (2013) and later modified by X. Zhou et al. (2017b). The scale has 20 items with four subscales corresponding to four symptom clusters: intrusive, hyperarousal, avoidance, and negative alterations in cognition and mood. A 5-point rating system was used for responses, where 0 indicated “not at all” and 4 indicated “almost every day”. Elevated total scores are indicative of more severe PTSD among adolescents. Cronbach’s alpha was 0.92 for this part of the study.

#### 2.2.5. PTG

To evaluate PTG among adolescents, the Chinese version of the Posttraumatic Growth Inventory (PTGI), created by Tedeschi and Calhoun (1996) and translated by X. Zhou et al. (2014), was employed. This scale assesses positive changes in self-perception, interpersonal relationships, and life philosophy through subscales, encompassing a total of 22 items. A 6-point rating system was used for responses, where 0 indicated “no change” and 5 indicated “changed a lot”. Higher total scores indicate more pronounced positive changes following the public emergency and higher levels of PTG. This part of the research showed a Cronbach’s alpha of 0.94.

### 2.3. Data Analysis

Descriptive statistics and model testing were carried out using SPSS 21.0 and Mplus 8.3. Descriptive statistics (means and SD), intercorrelations, and demographic differences in the main variables were all generated using SPSS. A three-step procedure was then employed in Mplus for model analysis. Analysis commenced with testing the direct impact of social support on PTG and PTSD while controlling for demographic variables (only-child status, grade, gender). Building upon this direct model, a mediation model was formulated by introducing empathy, PCSs, and NCSs as proposed mediators. Specific pathways were drawn from empathy to both PCSs and NCSs. The last phase constrained non-significant pathways to zero, resulting in a refined and parsimonious path model. During model analysis, we evaluated model fit using various indices: CFI, TLI, chi-square, SRMR, and RMSEA. The cutoff values for model acceptance were TLI and CFI values greater than 0.90 and SRMR and RMSEA values less than 0.08.

To assess the potential impact of shared method variance due to the use of self-report measures, we conducted Harman’s single-factor test (Podsakoff & Organ, 1986). The results of unrotated exploratory factor analysis indicated that there were 18 factors with eigenvalues greater than 1, accounting for 62.36% of the total variance. The first factor explained 20.55% of the variance, which is below the critical value of 40%. These results suggest that shared method variance is unlikely to be a serious concern in the present study.

## 3. Results

### 3.1. Preliminary Analyses

First, this study examined demographic differences in social support, empathy, PCSs, NCSs, PTG, and PTSD (see Supplementary Table S1). The results revealed significant gender differences in social support, empathy, and PTSD. Additionally, there were significant differences in social support and NCSs between students in grades 7 and 8, respectively. PCSs and PTG scores also differed significantly depending on whether adolescents were only children or not. Non-only child participants reported significantly higher levels of PCSs and PTG compared with their only-child counterparts. Consequently, only-child status, grade, and gender were entered into subsequent models as control factors.

Table 1 displays the correlations between social support, empathy, PCSs, NCSs, PTG, and PTSD. Analyses indicated that social support correlated negatively with NCSs and PTSD, but positively with empathy, PCSs, and PTG. Empathy was negatively related to PTSD but positively related to both PTG and PCSs. PCSs correlated positively with PTG but negatively with PTSD. In contrast, NCSs were positively correlated with both PTSD and PTG. Furthermore, a positive correlation existed between PCSs and NCSs. Meanwhile, PTSD and PTG were inversely correlated.

### 3.2. Examination of the Mediation Model

Prior to testing the mediation hypotheses, a direct effects model (see Supplementary Figure S1) was specified to evaluate the links connecting social support with PTG and PTSD, after controlling for only-child status, grade, and gender. The direct effects model fit the data well, χ^2^_(53)_ = 317.290, TLI = 0.928, CFI = 0.949, RMSEA (90% CI) = 0.075 (0.067–0.083), SRMR = 0.051. Examination of the path coefficients indicated that social support was correlated positively with PTG (*β* = 0.514, *p* < 0.001) and negatively with PTSD (*β* = −0.362, *p* < 0.001).

Next, the direct effects model was extended by incorporating empathy, PCSs, and NCSs as potential mediators in the relationship between social support and PTG/PTSD (see Supplementary Figure S2). Pathways from empathy to both PCSs and NCSs were specified, culminating in a mediation model. The mediation model showed good fit to the data, χ^2^_(74)_ = 372.315, TLI = 0.923, CFI = 0.951, RMSEA (90% CI) = 0.068 (0.061–0.075), SRMR = 0.046. The analysis identified several statistically significant paths: from social support to PCSs, NCSs, empathy, PTG, and PTSD; from empathy to PCSs and PTG; from PCSs to PTG and PTSD; and from NCSs to PTG and PTSD. No other paths proved to be significant.

**Figure 2:**
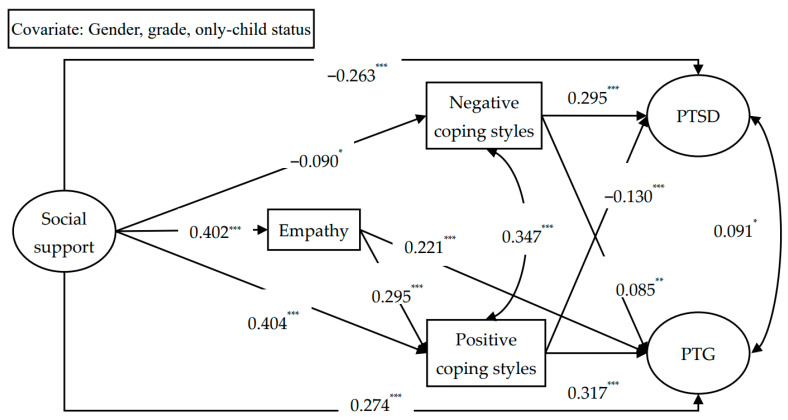
Parsimonious paths model. Note: * *p* < 0.05, ** *p* < 0.01, *** *p* < 0.001; PTSD = posttraumatic stress disorder, PTG = posttraumatic growth.

We fixed all non-significant paths to zero and specified a more parsimonious path model (Figure 2). The analysis revealed that the model fitted the data well, χ^2^_(88)_ = 390.459, TLI = 0.935, CFI = 0.951, RMSEA (90% CI) = 0.063 (0.056–0.069), SRMR = 0.048. Social support showed positive links with empathy, PCSs, and PTG, while its association with NCSs was negative. Empathy exhibited positive relations with both PTG and PCSs. Additionally, both PCSs and NCSs showed positive associations with PTG. In summary, social support related to PTG both directly and through four mediational routes: three 1-step paths via empathy, PCSs, and NCSs, one two-step path from empathy to PCSs. Furthermore, social support and PCSs showed negative relations with PTSD, whereas NCSs showed a positive association. Thus, social support negatively related to PTSD both directly and through three mediational routes: two one-step paths via PCSs and NCSs and one two-step path from empathy to PCSs.

To examine the significance of the indirect effects, a bias-adjusted bootstrap procedure was employed. A significant indirect effect is inferred if the 95% CI for its estimate excludes 0. As presented in Table 2, the bootstrap results revealed that for all seven previously mentioned indirect paths, the 95% CI excluded zero, providing further evidence of the validity of the aforementioned mediations.

## 4. Discussion

This study investigated the simultaneous pathways linking social support to PTSD and PTG and examined differentiated indirect pathways involving empathy and coping styles among adolescents. Consistent with prior evidence, social support showed the expected pattern, being negatively associated with PTSD and positively associated with PTG. Importantly, the present study extends this literature by delineating differential indirect pathways within a model: empathy mediated the association between social support and PTG but not PTSD; PCSs operated as a shared pathway (associated with lower PTSD and higher PTG); and NCSs showed a double-edged role (positively associated with both PTSD and PTG). These results suggest mechanistic differentiation between PTSD and PTG, aligning with prior work (X. Zhou et al., 2022). These findings provide a more fine-grained test of the three-phase process model (X. Zhou & Zhen, 2024) and COR theory (Hobfoll, 2011) by specifying which cognitive, affective and coping-related resources may be more relevant to distress versus growth in adolescents during a public health emergency.

Specifically, a direct positive association was observed between social support and PTG, while an inverse relationship was found with PTSD, aligning with earlier evidence (An et al., 2018). These patterns accord with COR theory (Hobfoll, 2011), indicating that social support is a key external resource aiding posttraumatic adjustment. For adolescents, social support can alleviate the resource depletion caused by trauma and reduce negative emotions (Wu et al., 2016), as well as reduce their PTSD (Zalta et al., 2021). Social support expands social and psychological resources for coping with traumatic events, encourages adolescents to have positive thoughts about traumatic events, and facilitates closer interpersonal connections (Feeney & Collins, 2015), subsequently promoting positive change and fostering PTG development.

Analysis revealed a positive association between social support and PTG via empathy. However, no such significant pathway was identified for PTSD. During a public health emergency, adequate social support helped adolescents maintain healthy interpersonal relationships with others, and made them more willing to understand the emotions and thoughts of others. As their perceived social support increased, their tendency toward healthier relationships and thus empathy towards others also increased (Van Heel et al., 2020). Consequently, social support serves to bolster empathy in adolescents (X. Wang et al., 2022), which in turn helps them view traumatic events from multiple perspectives, prompts adolescents to rethink the meaning of traumatic events, helps them explore positive changes after a public health emergency, and ultimately fosters PTG. The literature provides converging support for this perspective (Jambon et al., 2019). However, our analysis did not support a mediating effect of social support on PTSD via empathy. One possible reason for this result is that although empathy can enable adolescents to resonate emotionally and cognitively with others, this resonance is often limited. PTSD is a highly complex and long-lasting negative psychological reaction, and it is difficult for cross-sectional research to capture alleviating effects of limited resonance through empathy. Additionally, empathy consists of both cognitive and emotional components, which may play distinct roles in trauma-related outcomes. In this study, empathy was treated as a general construct without distinguishing its cognitive and emotional components, which may explain why empathy did not exert a significant effect on PTSD, and could be further explored in future research to better understand their differential roles in PTSD and PTG. Therefore, in the current study, no significant path from empathy to PTSD was observed, and social support showed no indirect association with PTSD via empathy.

Analysis also indicated an inverse relationship between social support and PTSD, as well as a positive relationship with PTG mediated by PCSs, confirming our initial hypothesis. Cohen and Wills (1985) suggested that sufficient social support can enhance individuals’ coping abilities, and individuals perceiving strong support tend to use bravery and PCSs when facing traumatic events (L. Davis & Brekke, 2014; L. Zhang et al., 2021). For adolescents, social support may promote the ability to adopt PCSs, thereby facilitating their cognitive restructuring and understanding of the public health emergency. For example, adolescents may increase engagement with family and friends, strengthen bonds, and identify additional ways to relieve stress. Therefore, PCSs can not only help adolescents recognize the positive changes in their lives, but also help them better handle the negative consequences of the public health emergency and reduce the impact of negative emotions, ultimately alleviating their PTSD (Vernon et al., 2009) and promoting PTG (Peters et al., 2021).

A notable and somewhat unexpected finding was that social support negatively related to both PTSD and PTG via NCSs, a result that was not fully consistent with Hypothesis 2. Generally, a lack of adequate social support is associated with a greater propensity for using NCSs, like avoidance and complaining (Liu et al., 2016; L. Zhang et al., 2021), which can have adverse consequences for both physical and psychological well-being. However, despite being classified as NCSs, these strategies may sometimes serve as methods adopted by individuals to achieve positive outcomes (Guo et al., 2017). Specifically, for adolescents, although the effect is minimal, NCSs contribute to PTG development, as they may serve as a useful way to cope with trauma in the short term. This phenomenon is not unique to adolescents, as analogous findings have been reported in research with other demographic groups (Ai et al., 2022; Fu et al., 2022). However, it is important to recognize that PTG, in some cases, may reflect illusory forms of growth (Boals, 2023); studies conducted during the COVID-19 pandemic also find this (Park et al., 2023). These forms of growth might provide temporary emotional relief or an illusory sense of control, but they do not necessarily reflect genuine personal development. Therefore, while NCSs might offer some immediate coping benefits, NCSs often do not resolve underlying issues, and they likely exacerbate the deleterious effects of trauma and distress on mental health (Du et al., 2018), contributing to increased PTSD. Collectively, these results point to a double-edged role of NCSs in the context of a public health crisis like a pandemic: while NCSs may offer short-term benefits, they carry significantly greater potential for harm.

Social support was also negatively associated with PTSD and positively associated with PTG through multiple mediating effects of empathy via PCSs. During a public health emergency, adequate social support can help adolescents improve their empathy (X. Wang et al., 2022), enabling them to understand the public emergency from a more comprehensive perspective, inclining them toward constructive PCSs (Noda et al., 2018). These coping styles help alleviate adolescents’ anxious and maladaptive emotional states while also buffering against the effects of negative affect (Liao et al., 2022). Furthermore, they provide opportunities for adolescents to engage in rethinking traumatic events, which may help them reshape their world assumptions through assimilation or accommodation (Wu et al., 2018). Specifically, assimilation involves integrating new traumatic experiences into existing belief systems without changing core assumptions, while accommodation involves adjusting or altering these core beliefs to better fit new experiences. These processes might help adolescents reduce PTSD symptoms. Additionally, they may also protect adolescents from further psychological harm or facilitate recovery from PTSD and promote PTG.

The current results revealed that the path from empathy to NCSs was non-significant. This finding may be attributed to the unique development of coping styles in adolescent populations. Adolescents are in a period of rapid development of coping styles (Skinner & Zimmer-Gembeck, 2007), and the coping styles they adopt are often learned through education, observation (Williams & McGillicuddy-De Lisi, 1999) and interaction with caregivers and peers. Our investigation found a lower incidence of NCSs use in the adolescent cohort, and it is unlikely that schools would play a significant role in teaching these NCSs. Consequently, even among highly empathic adolescents, it is unlikely that empathy would lead them to adopt NCSs. Therefore, empathy did not exert a substantial influence on NCSs in this adolescent sample, and the hypothesized indirect pathway from social support to PTSD/PTG through empathy and then NCSs was not supported.

The present study has several limitations. The first limitation concerns our methodological dependence on self-report questionnaires, which may introduce reporting biases, such as distortions related to current affective state and social desirability. Future research should incorporate standardized interviews, experiments, or other objective methodologies to reduce shared method variance and enhance the reliability of the findings. Second, public health emergencies like the COVID-19 pandemic vary greatly in severity and duration, and adolescents’ experiences are shaped by a range of factors, including family resources, perceived threat, and prior vulnerabilities. In this study, these contextual factors were not measured, which constrains the interpretation of results. Although current findings are valid within the scope of this sample, future studies should explore these variables to gain a deeper understanding of how social support functions in different contexts. Second, this study was conducted among Chinese adolescents during the COVID-19 pandemic. Public health emergencies differ in severity and duration, and adolescents’ experiences may also depend on contextual conditions, such as family resources, perceived threat, and prior vulnerabilities. These factors were not measured in the present study, so interpretation of the observed associations is constrained. Moreover, cultural norms around social support and coping styles may shape these relationships; thus, generalization to other cultural contexts should be cautious. Future research should incorporate these contextual indicators and conduct cross-cultural replications. A further limitation concerns empathy measurement. We assessed empathy using only the perspective-taking and empathic concern subscales of IRI (α = 0.71), excluding fantasy and personal distress, which may lead to different coping outcomes. This narrower operationalization and the modest reliability may have attenuated the results in this study, which should be considered when interpreting these findings. Future studies could include all IRI subscales or examine empathy components separately. Moreover, because of the cross-sectional nature of this study, the observed associations should be interpreted as correlational rather than causal. Prospective studies employing longitudinal or experimental methodologies are needed to probe the potential mechanisms between social support, empathy, coping styles, and posttraumatic outcomes.

Notwithstanding these limitations, this investigation offered insights into the unique or shared pathways underlying PTSD and PTG among adolescents experiencing a public health crisis, as viewed through the lens of external resources. The findings indicate that social support plays a consistently beneficial role, being associated with lower PTSD and higher PTG among adolescents. However, the findings suggest that the potential underlying mechanisms of PTG and PTSD are different, with empathy, PCSs, and NCSs playing different roles. These findings enrich theoretical research that examines the COR theory and the three-phase process model (Hobfoll, 2011; X. Zhou & Zhen, 2024), expanding the application scope of these theories. Regarding intervention practice, the current findings suggest that practitioners in the mental health field should highlight the value of social support in helping adolescents cope with traumatic events to parents, teachers, and wider society. We should also recognize the potential roles of empathy and different coping styles, and encourage teachers and parents to guide adolescents in developing appropriate empathy skills, solving problems with PCSs, and promoting the healthy psychological development of adolescents after trauma.

## Figures and Tables

**Table 1:** Descriptive statistics and correlations among main variables.

Variables	*M* (*SD*)	1	2	3	4	5
1. Social support	55.68 (15.69)	-				
2. Empathy	48.24 (7.48)	0.38 **	-			
3. PCSs	28.75 (7.45)	0.49 **	0.44 **	-		
4. NCSs	15.58 (4.62)	−0.07 *	−0.06	0.23 **	-	
5. PTSD	25.95 (16.16)	−0.36 **	−0.16 **	−0.16 **	0.36 **	-
6. PTG	56.41 (24.22)	0.48 **	0.43 **	0.56 **	0.12 **	−0.11 **

Note: * *p* < 0.05, ** *p* < 0.01; PCSs = positive coping styles, NCSs = negative coping styles, PTSD = posttraumatic stress disorder, PTG = posttraumatic growth.

**Table 2:** Bias-corrected bootstrap test of mediating effects.

Paths	Estimate	95% Confidence Interval	
		Lower	Upper
Social support—PTSD	−0.267	−0.359	−0.173
Social support—NCSs—PTSD	−0.041	−0.075	−0.011
Social support—PCSs—PTSD	−0.052	−0.092	−0.020
Social support—empathy—PCSs—PTSD	−0.015	−0.028	−0.006
Social support—PTG	0.271	0.181	0.362
Social support—empathy—PTG	0.099	0.069	0.135
Social support—NCSs—PTG	−0.010	−0.025	−0.002
Social support—PCSs—PTG	0.120	0.079	0.164
Social support—empathy—PCSs—PTG	0.035	0.023	0.051

Note: PCSs = positive coping styles, NCSs = negative coping styles, PTSD = posttraumatic stress disorder, PTG = posttraumatic growth.

## Data Availability

The data presented in this study are available on request from the corresponding author due to the terms of the informed consent agreement, which states that the raw data is only accessible to the research team.

## Supplementary Materials

The following supporting information can be downloaded at https://www.mdpi.com/article/10.3390/bs16030377/s1, Figure S1: The direct effects model between social support, PTSD and PTG; Figure S2: The indirect model of empathy and coping styles between social support, PTSD and PTG; Table S1: Demographic differences in main variables.
