# Supplementary material for: Social Support, Posttraumatic Stress Disorder and Growth Among Adolescents During Public Health Emergencies: Mediating Roles of Empathy and Coping Styles
Source: Behav Sci (Basel). 2026 Mar 6;16(3):377. doi: 10.3390/bs16030377 (PMC13023649; doi:10.3390/bs16030377)
Supplement: Supplementary file 1 [file behavsci-16-00377-s001.zip › behavsci-4042238-supplementary.pdf]

Supplementary Material

Table S1. Demographic differences in main variables

Variables	Gender		Grade		Only-child status	
	t	*p*	*t*	*p*	*t*	*p*
1. Social support	**2.289**	**0.022**	**2.528**	**0.012**	-0.814	0.416
2. Empathy	**5.335**	**<0.001**	-0.496	0.620	-0.304	0.761
3. PCS	-0.082	0.935	0.772	0.440	**-2.024**	**0.043**
4. NCS	-1.274	0.203	**-3.982**	**<0.001**	-0.778	0.437
5. PTSD	**-4.377**	**<0.001**	-1.620	0.106	0.782	0.435
6. PTG	1.747	0.081	1.344	0.179	**-2.313**	**0.021**

Note. PCS = positive coping styles, NCS = negative coping styles, PTSD = posttraumatic stress disorder, PTG = posttraumatic growth.

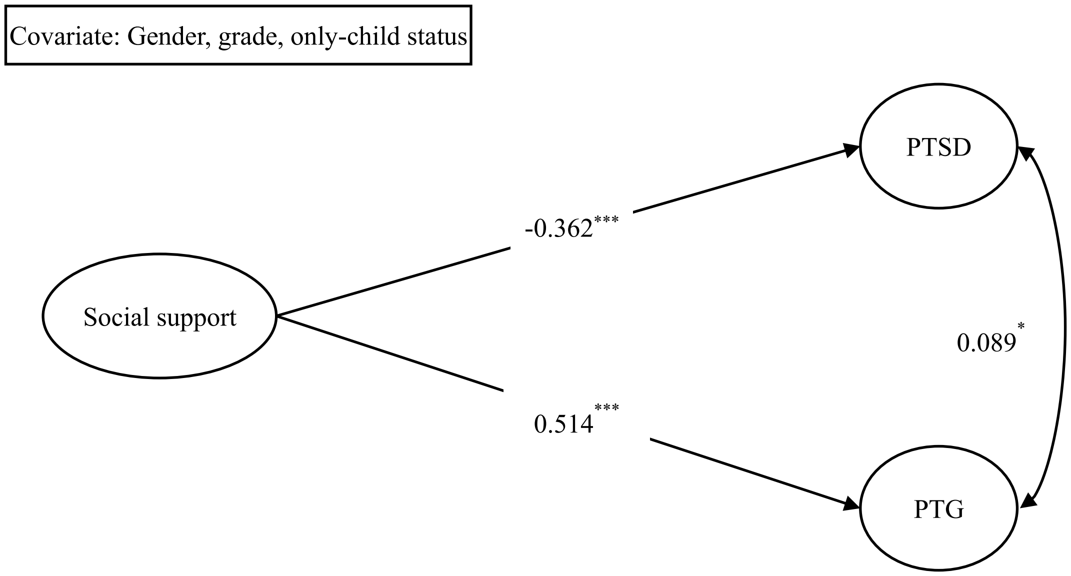

Figure S1. The direct effects model between social support, PTSD and PTG

Note. *$p < 0.05$, ***$p < 0.001$; PTSD = posttraumatic stress disorder, PTG = posttraumatic growth.

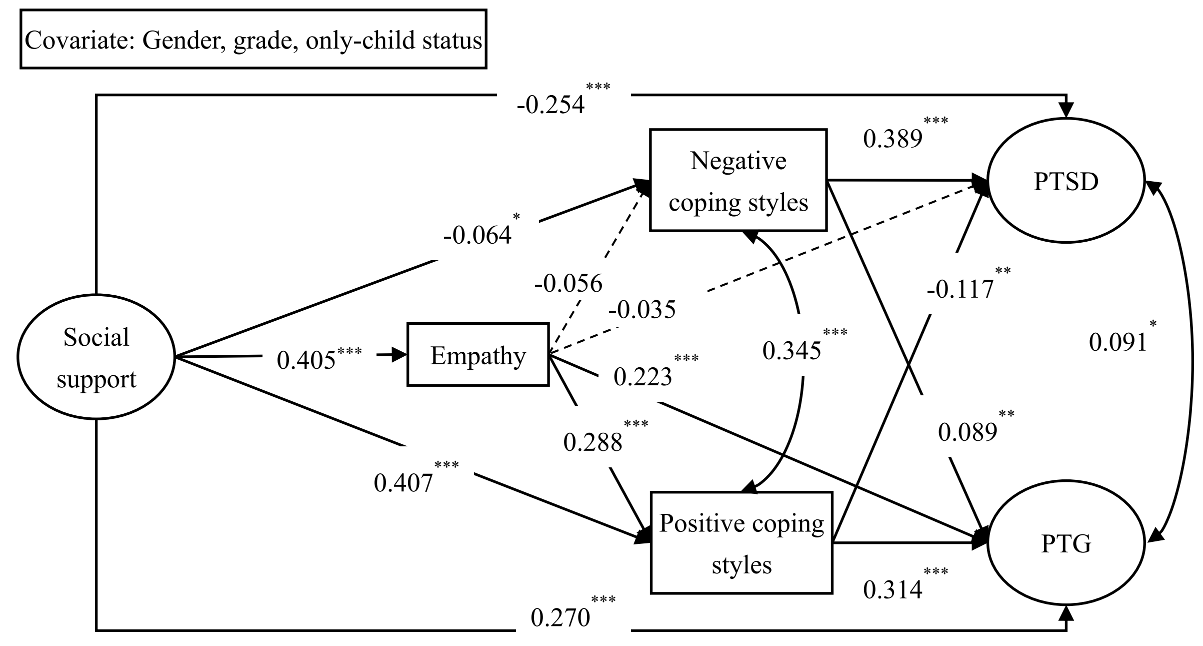

Figure S2. The indirect model of empathy and coping styles between social support, PTSD and PTG

Note. **p* < 0.05, ***p* < 0.05, ****p* < 0.001; PTSD = posttraumatic stress disorder, PTG = posttraumatic growth.
